# Supplementary material for: Tetrameric architecture of an active phenol-bound form of the AAA+ transcriptional regulator DmpR
Source: Nat Commun. 2020 Jun 1;11:2728. doi: 10.1038/s41467-020-16562-5 (PMC7264223; doi:10.1038/s41467-020-16562-5)
Supplement: Supplementary file 1 — Supplementary Information [file 41467_2020_16562_MOESM1_ESM.pdf]

Supplementary Information

Tetrameric architecture of an active phenol-bound form of the AAA$^+$ transcriptional regulator DmpR

Kwang-Hyun Park, Sungchul Kim *et al.*

Supplementary Figure

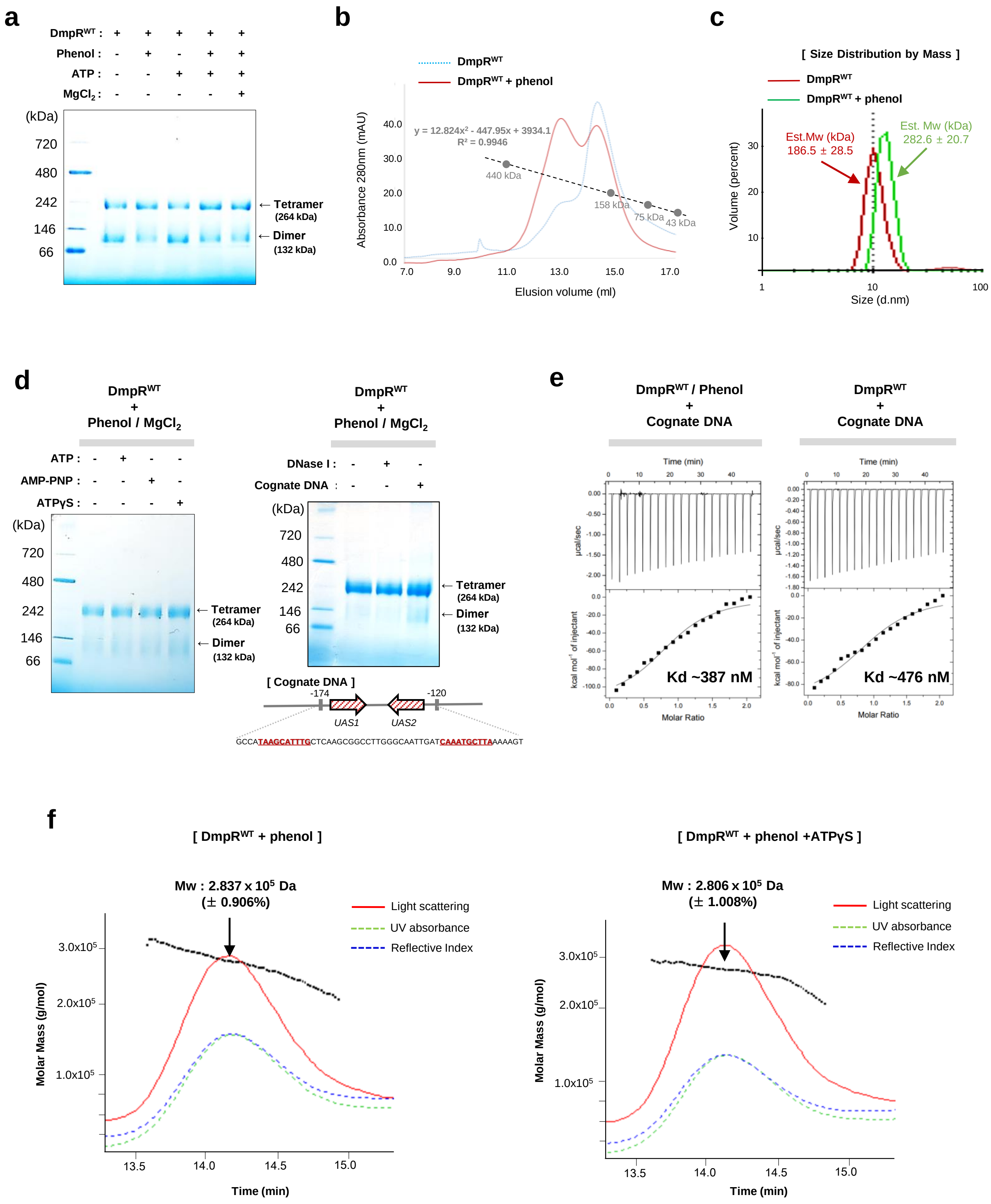

Supplementary Figure 1. Analysis of DmpR oligomers.

(a) BN-PAGE analysis of N-terminally His-tagged $DmpR^{WT}$ in the presence or absence of phenol. This data is representative of three replicates with similar results.

(b) Size exclusion chromatography of $DmpR^{WT}$. The dotted blue lines and the red lines correspond to the elution profile of the $DmpR^{WT}$ in the absence or presence of the phenol, respectively.

(c) Dynamic light scattering of $DmpR^{WT}$. The red lines and the green lines correspond to the mass distribution of the $DmpR^{WT}$ in the absence or presence of the phenol, respectively.

(d) Effect of ATP analogues and cognate DNA for specific binding by DmpR on the oligomerization of $DmpR^{WT}$. This data is representative of three replicates with similar results.

(e) ITC analysis of the binding of cognate DNA to the $DmpR^{WT}$ in the presence (left) and absence (right) of phenol

(f) MALS profiles of $DmpR^{WT}$ in the presence of phenol (left) and in the presence of phenol and ATPγS (right).

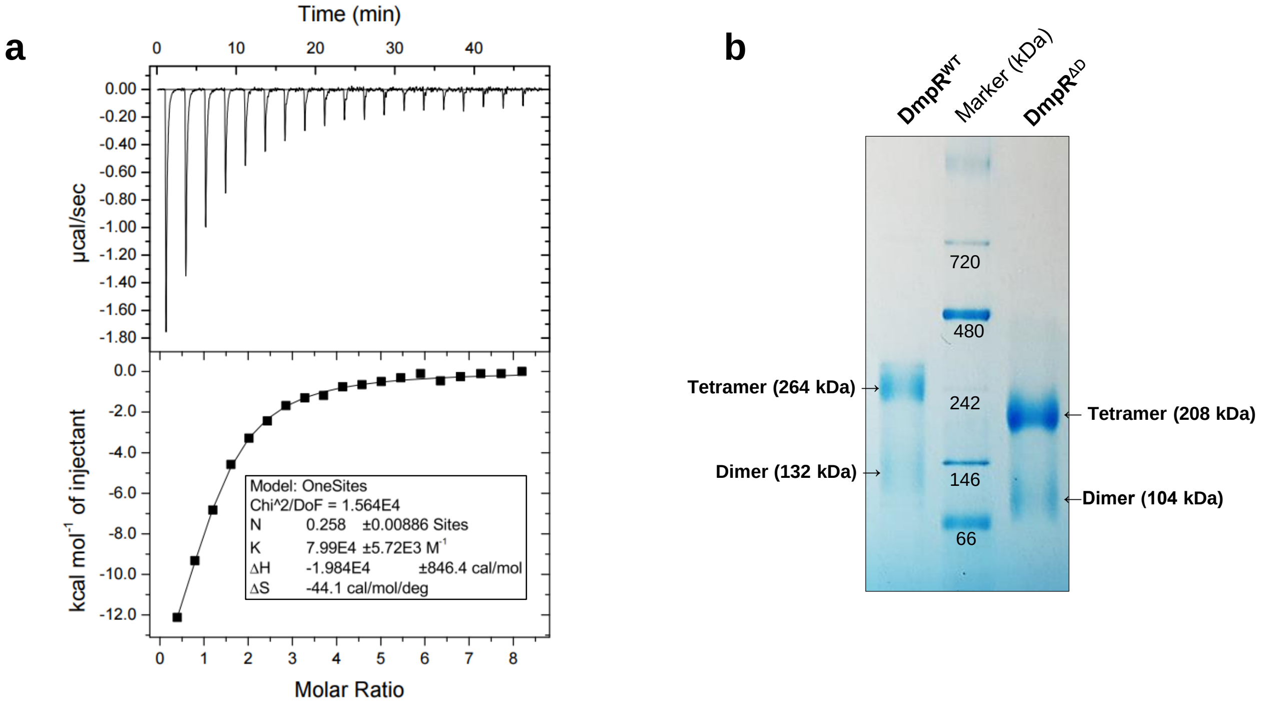

Supplementary Figure 2. Characterization of purified DmpR$^{\Delta D}$.

(a) ITC analysis of phenol binding by DmpR$^{\Delta D}$.

(b) BN-PAGE analysis of DmpR^{WT} and DmpR$^{\Delta D}$. The corresponding expected molecular weights are indicated. This data is representative of three replicates with similar results.

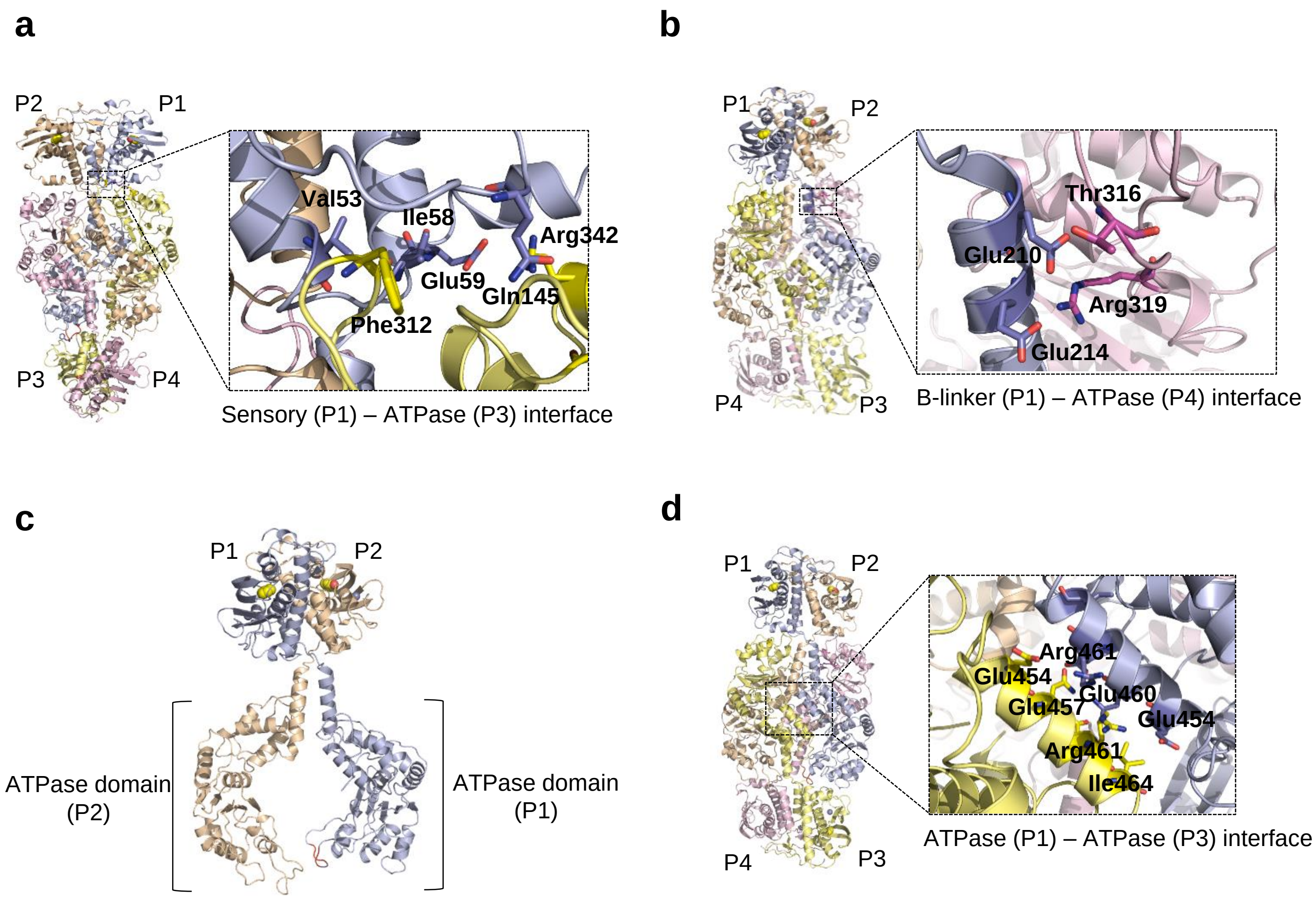

Supplementary Figure 3. Interfaces within the tetrameric structure.

(a) The interface between the sensory and ATPase domains is shown within the enlarged box, with residues involved in hydrophobic and charged interactions in stick representation (P1, light blue; P3, yellow).

(b) Residues involved in the interface between the B-linker and the ATPase domain (P1, light blue; P4, light pink).

(c) The dimeric structure of the head-to-head dimer of P1 (light blue) and P2 (pink), illustrating the juxtaposition of the two separate ATPase domains.

(d) Residues involved in the interface between the ATPase domains of P1 (light blue) and P3 (pale yellow).

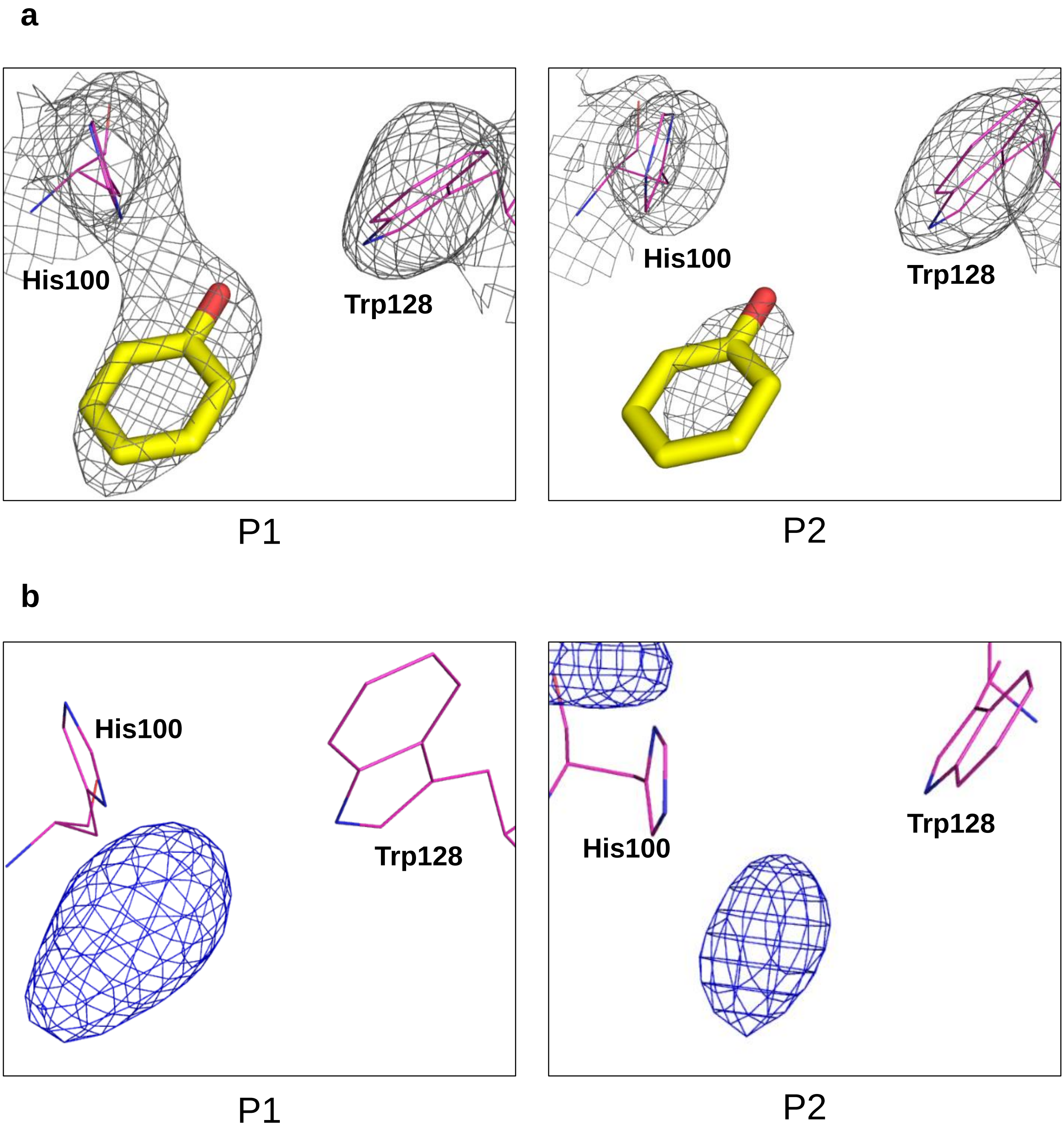

Supplementary Figure 4. Different electron densities of phenol in the P1/P2 dimer.

(a) The 2mFo-DFc map (1.5σ) shows strong electron density for the phenol ligand in the sensory domain of P1 (left). The 2mFo-DFc map (1.5σ) shows weak electron density for the phenol ligand in the sensory domain of P2 (right).

(b) The mFo-DFc omit map (2.5σ) of the sensory domain of P1 (left). The mFo-DFc omit map (2.5σ) of the sensory domain of P2 (right).

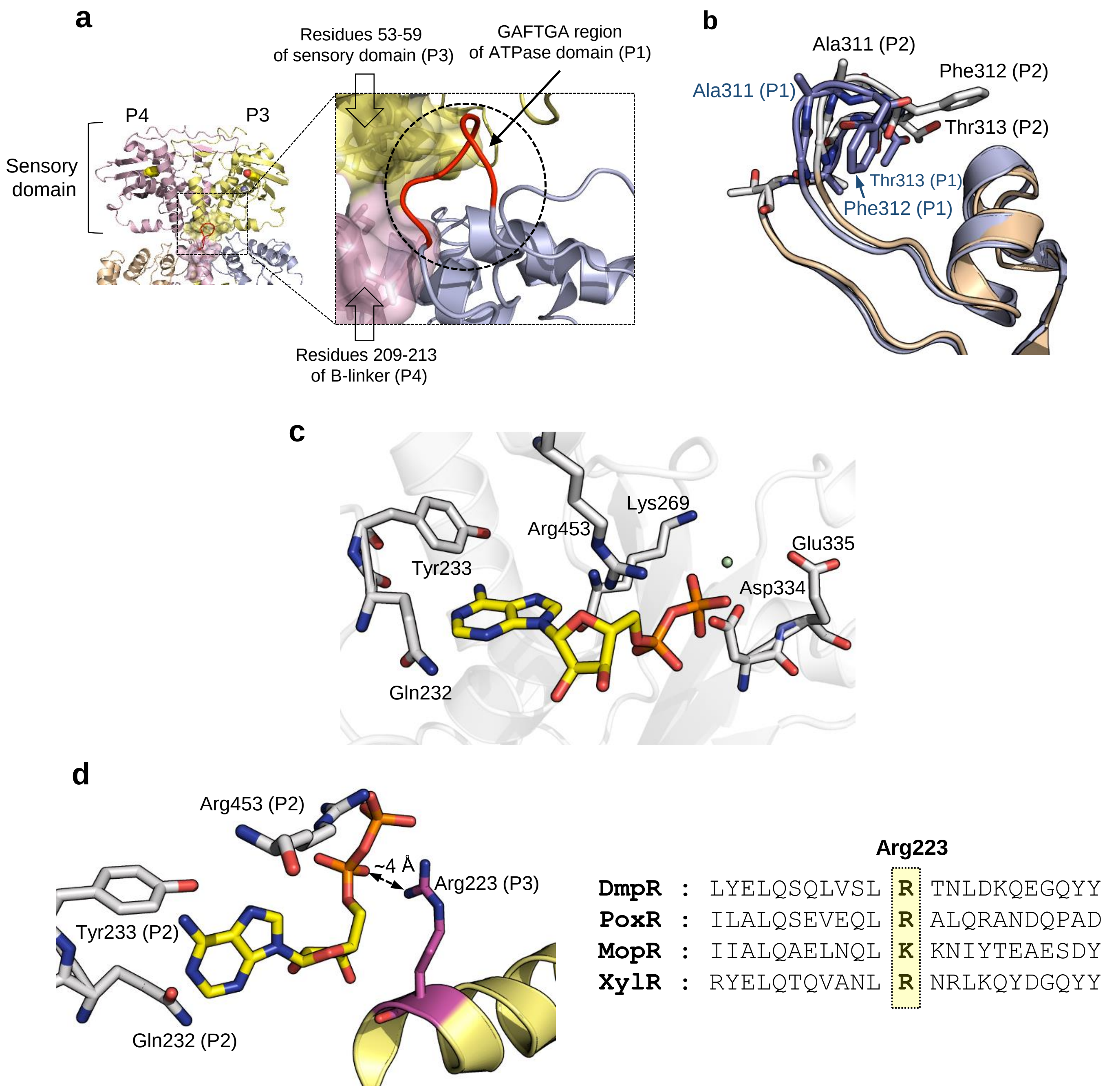

Supplementary Figure 5. Structure of the ATPase domain.

(a) Interaction of the sensory domain (P3, pale yellow surface) and GAFTGA region (P1, red cartoon) in the ATPase domain within the tetrameric complex.

(b) Superimposition of the ATPase domains of P1 and P2 to highlight the variations in the GAFTGA region.

(c) Nucleotide binding site and a hypothetical ADP-bound model of the ATPase domain. Key residues interacting with the nucleotide are shown as sticks.

(d) Arg223 is located in proximity to the ATP binding site within the tetrameric complex, as highlighted by the stick representations (left). Sequence alignment of four aromatic effector-binding transcriptional activators (right). The box indicates the conservation of the arginine residue in the B-linker helix.

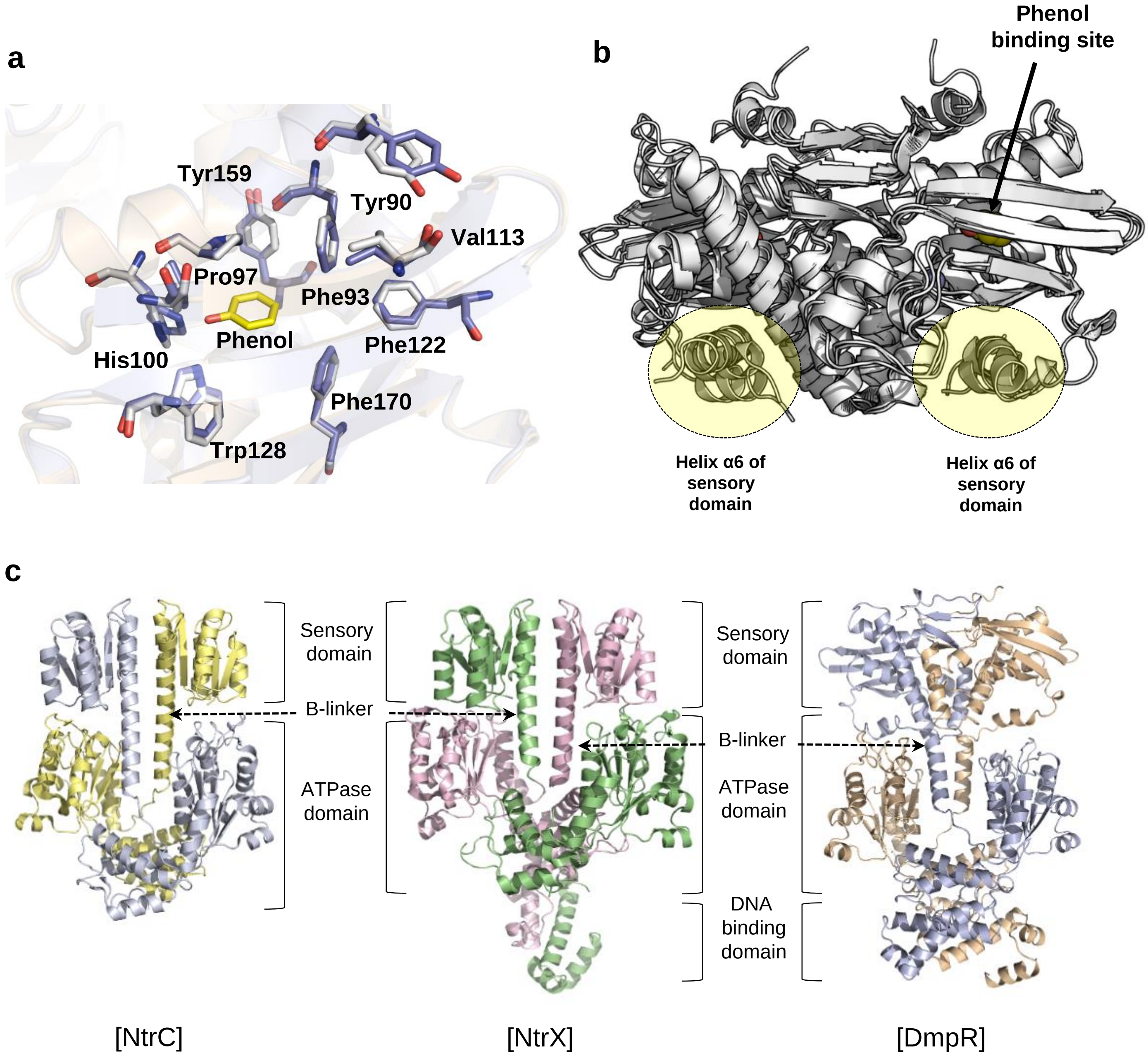

Supplementary Figure 6. Conformational changes of the sensory domain

(a) Movement of residues in the phenol binding site. Changes in each residue are indicated by overlaid stick representations (blue for the P1 protomer and white for the P2 protomer).

(b) Structural flexibility of helix α6 in the sensory domain. Superimposition of the sensory domains of MopR (PDB ID, 5kbi), PoxR (PDB ID, 5fru) and DmpR is represented. Helix α6, which has high structural flexibility, is indicated by yellow circles.

(c) Inactive dimer model of DmpR (right) based on the NtrC1 dimer (PDB ID, 1ny5, left) and NtrX dimer (PDB ID, 5m7o, centre) structures. The DNA binding domain of DmpR was constructed based on the Fis protein (PDB ID, 4fis, sequence homology ~32%).

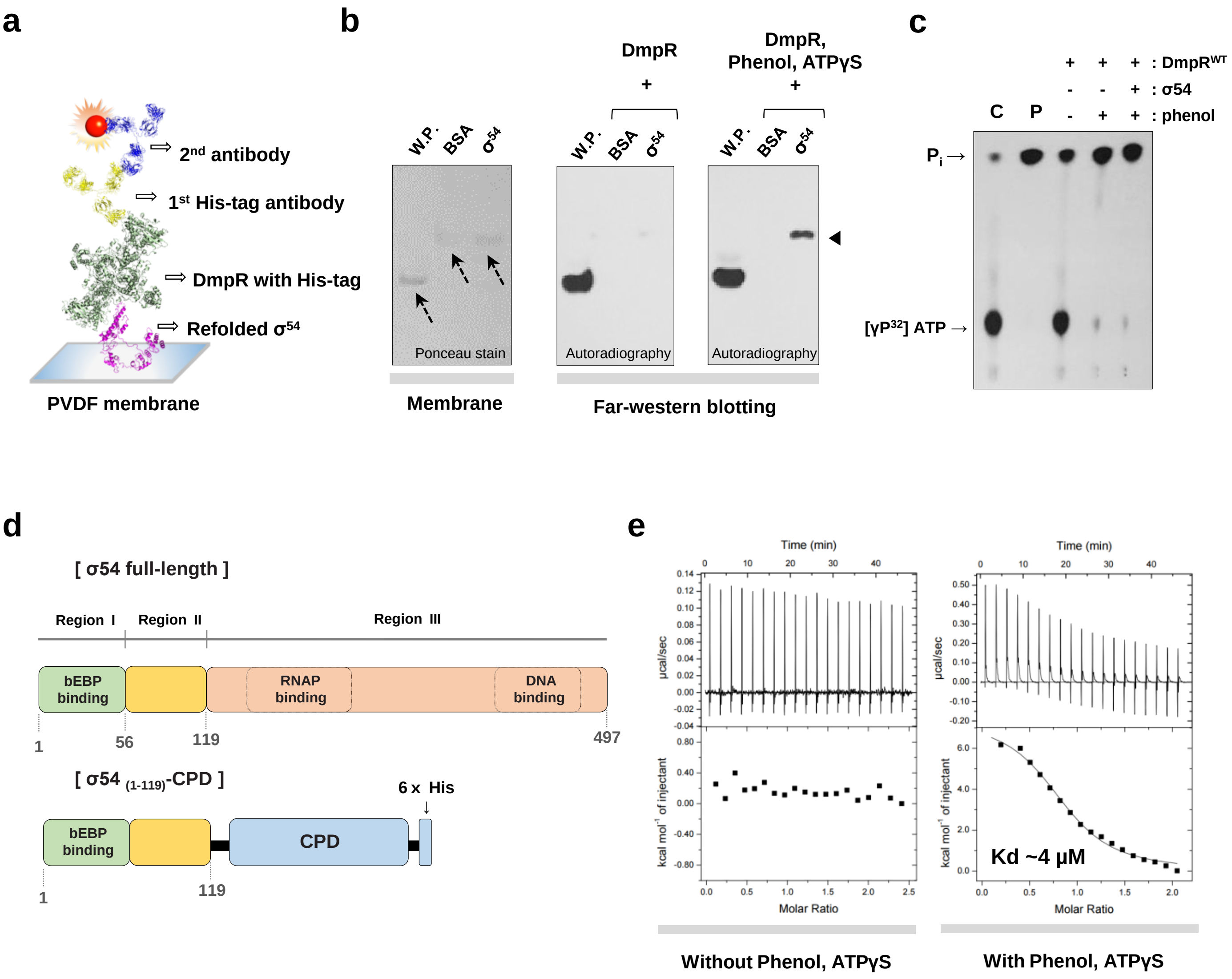

Supplementary Figure 7. Analysis of the interaction of DmpR with σ^{54}

(a) Schematic representation of the far-western blot visualization of $DmpR^{WT}$ bound to σ^{54}.

(b) Far-western blotting of $DmpR^{WT}$ and σ^{54}. 'W.P.' indicates a western blot positive control reaction. 'BSA' indicates a far-western blot negative control reaction. All data are representative of five replicates with similar results.

(c) ATPase activity of $DmpR^{WT}$ in the presence or absence of σ^{54}. Hydrolysis of [γ-P^{32}] ATP to generate P^{32} was visualized by thin-layer chromatography. 'C' represents the reaction with the reaction buffer as control. 'P' represents the reaction with the alkaline phosphatase as positive control. This data is representative of three replicates with similar results.

(d) Schematic diagrams of wild type σ^{54} and truncated σ^{54} proteins.

(e) ITC analysis of the binding of $\sigma^{54}{}_{(1\text{-}119)}$-CPD to the $DmpR^{\mathbf{WT}}$ tetramer.

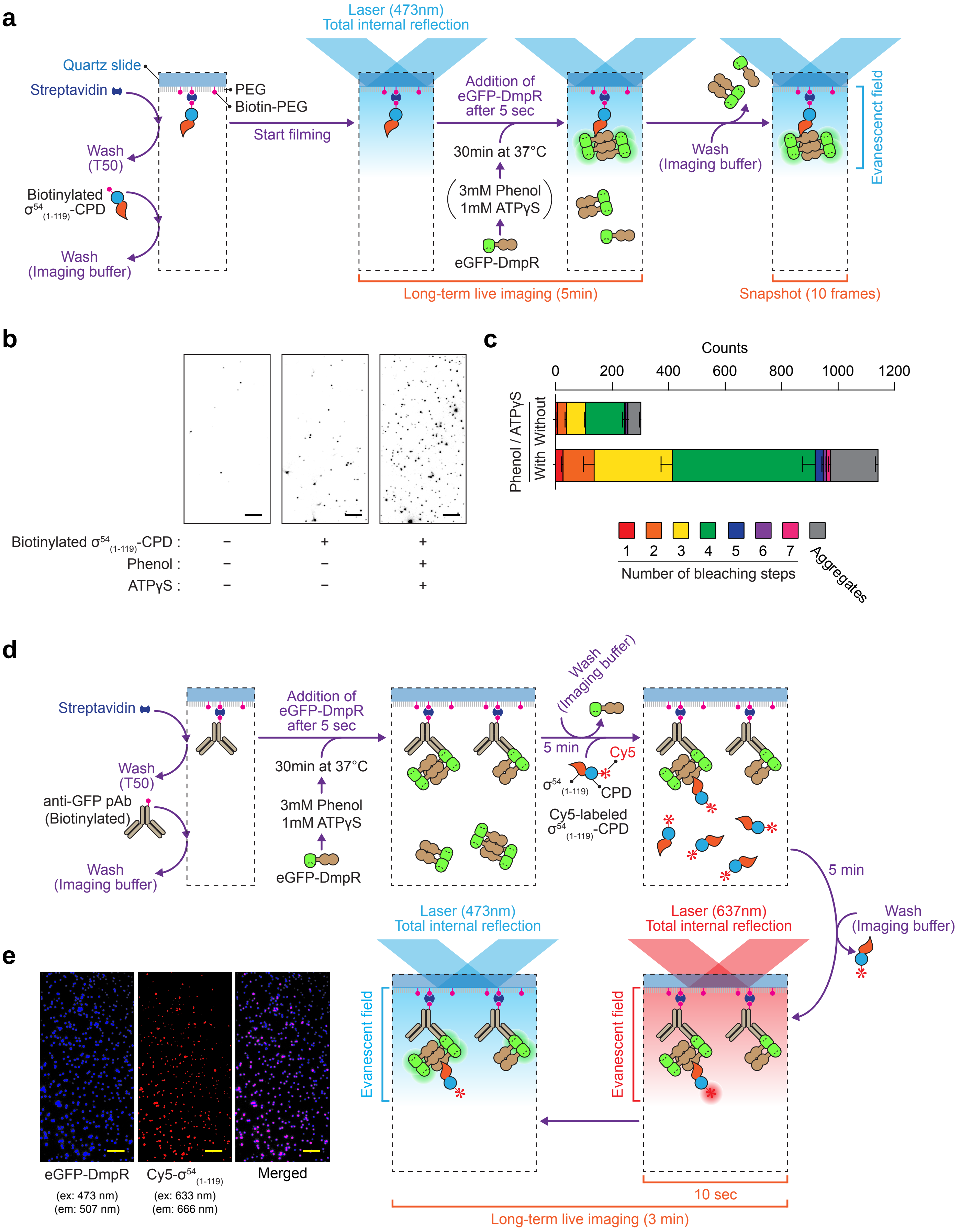

a
Laser (473nm)
Total internal reflection
Quartz slide
Streptavidin
PEG
Biotin-PEG
Wash
(T50)
Biotinylated
σ54(1-119)-CPD
Wash
(Imaging buffer)
Start filming
Addition of
eGFP-DmpR
after 5 sec
30min at 37°C
3mM Phenol
1mM ATPγS
eGFP-DmpR
Wash
(Imaging buffer)
Evanescent field
Long-term live imaging (5min)
Snapshot (10 frames)
b
Biotinylated σ54(1-119)-CPD : − + +
Phenol : − − +
ATPγS : − − +
c
Counts
0 200 400 600 800 1000 1200
Phenol / ATPγS
Without
With
1 2 3 4 5 6 7 Aggregates
Number of bleaching steps
d
Streptavidin
Wash
(T50)
anti-GFP pAb
(Biotinylated)
Wash
(Imaging buffer)
Addition of
eGFP-DmpR
after 5 sec
30min at 37°C
3mM Phenol
1mM ATPγS
eGFP-DmpR
Wash
(Imaging buffer)
5 min
Cy5
σ54(1-119) CPD
Cy5-labeled
σ54(1-119)-CPD
5 min
Wash
(Imaging buffer)
Laser (473nm)
Total internal reflection
Laser (637nm)
Total internal reflection
Evanescent field
Evanescent field
10 sec
Long-term live imaging (3 min)
e
eGFP-DmpR
(ex: 473 nm)
(em: 507 nm)
Cy5-σ54(1-119)
(ex: 633 nm)
(em: 666 nm)
Merged

Supplementary Figure 8. Single-molecule florescence imaging of the interaction of tetrameric DmpR with $\sigma^{54}_{(1\text{-}119)}$.

(a) Schematic workflow of a single-molecule TIRF assay of eGFP-DmpR binding to $\sigma^{54}_{(1\text{-}119)}$. $\sigma^{54}_{(1\text{-}119)}$, which was biotinylated at the CPD tag, was surface-immobilized onto a PEGylated quartz slide through the biotin-streptavidin interaction. The oligomeric states and the number of binding of eGFP-tagged DmpR were visualized by 473-nm laser excitation through long-term imaging and snapshot analysis, respectively.

(b) Representative CCD images of the snapshot analysis in (a). All data are representative of five replicates with similar results. Scale bars, 5 μm.

(c) Counts for the binding events of eGFP-DmpR without/with phenol and ATPγS during long-term imaging, as shown in panel (a). Each number of photobleaching steps is represented as a separate box with a different colour in the bar graphs . Data represent the mean ± SD from three independent experimental replicates .

(d) Illustrated workflow of a single-molecule TIRF assay of $\sigma^{54}_{(1\text{-}119)}$ binding to eGFP-DmpR. Biotinylated anti-GFP polyclonal antibodies (pAb) were surface-immobilized onto the PEGylated quartz slide through the biotin-streptavidin interaction. eGFP-DmpR complexes were subsequently tethered via antibody-antigen interactions. The oligomeric states of eGFP-tagged DmpR were visualized by 473-nm laser excitation in TIRF microscopy and by the Cy5 signals from the DmpR-bound Cy5-labelled $\sigma^{54}_{(1\text{-}119)}$-CPD by 633-nm laser excitation.

(e) Representative CCD images of the snapshot images of eGFP-DmpR (blue, left) and Cy5-labelled $\sigma^{54}_{(1\text{-}119)}$-CPD (red, center) and the merged image (right) with treatment of the biotinylated anti-GFP pAb in (d) All data are representative of five replicates with similar results. Scale bars, 5 μm.

Supplementary Table

Supplementary Table 1. Cloning primer information

Vector	Sequence
pProEx-Hta-DmpR^{WT}-For	TTTCAGGGCGCCATGGATCCGATGCCGATCAAGTAC
pProEx-Hta-DmpR^{WT}-Rev	TAGATTCGAAAGCGGCCGCTAGCCTTCGATGCC
pProEx-Hta-DmpR$^{\Delta D}$-For	TTTCAGGGCGCCATGGATCCGACCAACCTGATCCAC
pProEx-Hta-DmpR$^{\Delta D}$-Rev	TAGATTCGAAAGCGGCCGCTACGCCCGTGGGAACAG
pProEx-Hta-DmpR$^{\Delta S}$-For	TTTCAGGGCGCCATGGATCCGAACGACCCCATCATC
pProEx-Hta-DmpR$^{\Delta S}$-Rev	TAGATTCGAAAGCGGCCGCTAGCCTTCGATGCC
pProEx-Hta-DmpR^{BC}-For	TTTCAGGGCGCCATGGATCCGAACGACCCCATCATC
pProEx-Hta-DmpR^{BC}-Rev	TAGATTCGAAAGCGGCCGCTACGCCCGTGGGAACAG
pProEx-Hta-DmpR^{C}-For	TTTCAGGGCGCCATGGATCCGCAGTACTACGGCATC
pProEx-Hta-DmpR^{C}-Rev	TAGATTCGAAAGCGGCCGCTACGCCCGTGGGAACAG
pET22b-σ^{54}-For	TAAGAAGGAGATATACATATGAAACCATCGCTCGTCCTA
pET22b-σ^{54}-Rev	TATTTTTCCATCCGCAAGCTTCATCAGTCGCTTGCGTTC
pET22b-$\sigma^{54}{}_{(1\text{-}119)}$-For	TAAGAAGGAGATATACATATGAAACCATCGCTCGTCCTA
pET22b-$\sigma^{54}{}_{(1\text{-}119)}$-Rev	TATTTTTCCATCCGCAAGCTTCGGCAGGCTGCTGGCGCTGGTCTGGTA
pProEx-Hta-v1(mod)-LIC-DmpR-For	ATTGGATTGGAAGTACCGGGCGCCCTGAAAATACAGGTTTTCGG
pProEx-Hta-v1(mod)-LIC-DmpR-Rev	CCGAAAACCTGTATTTTCAGGGCGCC CGGTACTTCCAATCCAAT
pProEx-Hta-v1(mod)-LIC-DmpR-eGFP-For	TACTTCCAATCCAAT GTGAGCAAGGGCGAGGAGCTG
pProEx-Hta-v1(mod)-LIC-DmpR-eGFP-Rev	CTCTCGGCATGGACGAGCTGTACAAG GGCATTGGAAGTGGATTA
pProEx-Hta-v1(mod)-LIC-DmpR-eYFP-For	TACTTCCAATCCAAT GTGAGCAAGGGCGAGGAGC
pProEx-Hta-v1(mod)-LIC-DmpR-eYFP-Rev	CGGCATGGACGAGCTGTACAAG GGCATTGGAAGTGGATTA

Supplementary Table 2. Data collection and refinement statistics

	**DmpR-phenol complex**
**Data collection**	
Space group	$P2_12_12$
Cell dimensions	
a, b, c (Å)	178.64, 130.00, 110.28
α, β,γ (°)	90.00, 90.00, 90.00
Resolution (Å)	30.12-3.42 (3.54-3.42)*
R_{merge}	0.095 (0.675)
$I / \sigma I$	8.52 (1.20)
Completeness (%)	90.5 (65.6)
Redundancy	5.2 (5.0)
**Refinement**	
Resolution (Å)	3.42
No. reflections	31306
R_{work} / R_{free}	23.28/28.56
No. atoms	
Protein	14880
Ligand/ion	32
B-factors	91.53
Protein	91.60
Ligand/ion	62.75
R.m.s. deviations	
Bond lengths (Å)	0.003
Bond angles (°)	0.701

* Values in parentheses are for highest-resolution shell.
