# Supplementary material for: Tetrameric architecture of an active phenol-bound form of the AAA+ transcriptional regulator DmpR
Source: Nat Commun. 2020 Jun 1;11:2728. doi: 10.1038/s41467-020-16562-5 (PMC7264223; doi:10.1038/s41467-020-16562-5)
Supplement: Supplementary file 3 — Reporting Summary [file 41467_2020_16562_MOESM3_ESM.pdf]

nature research

Corresponding author(s): Eui-jeon Woo / Chirlmin Joo

Last updated by author(s): Apr 29, 2020

Reporting Summary

Nature Research wishes to improve the reproducibility of the work that we publish. This form provides structure for consistency and transparency in reporting. For further information on Nature Research policies, see Authors & Referees and the Editorial Policy Checklist .

Statistics

For all statistical analyses, confirm that the following items are present in the figure legend, table legend, main text, or Methods section.

n/a	Confirmed	
☐	☒	The exact sample size (*n*) for each experimental group/condition, given as a discrete number and unit of measurement
☐	☒	A statement on whether measurements were taken from distinct samples or whether the same sample was measured repeatedly
☐	☒	The statistical test(s) used AND whether they are one- or two-sided<br>*Only common tests should be described solely by name; describe more complex techniques in the Methods section.*
☐	☒	A description of all covariates tested
☒	☐	A description of any assumptions or corrections, such as tests of normality and adjustment for multiple comparisons
☐	☒	A full description of the statistical parameters including central tendency (e.g. means) or other basic estimates (e.g. regression coefficient) AND variation (e.g. standard deviation) or associated estimates of uncertainty (e.g. confidence intervals)
☒	☐	For null hypothesis testing, the test statistic (e.g. *F*, *t*, *r*) with confidence intervals, effect sizes, degrees of freedom and *P* value noted<br>*Give P values as exact values whenever suitable.*
☒	☐	For Bayesian analysis, information on the choice of priors and Markov chain Monte Carlo settings
☐	☒	For hierarchical and complex designs, identification of the appropriate level for tests and full reporting of outcomes
☐	☒	Estimates of effect sizes (e.g. Cohen's *d*, Pearson's *r*), indicating how they were calculated

Our web collection on statistics for biologists contains articles on many of the points above.

Software and code

Policy information about availability of computer code

Data collection	1. Protein crystal diffraction data were collected from Pohang Accelerator Laboratory 'MX7A beamline'. The collected diffraction data was processed using 'HKL2000'.<br>2. Single-molecule data was collected using a modified version of "SINGLE" software that was developed by the lab of Prof. Dr. Ha. The original version is available at: https://cplc.illinois.edu/software/.
Data analysis	1. 'CCP4 5.1', 'Phenix 1.14' and 'wincoot 0.8.9' were used for protein structure determination and analysis.<br>2. Molecular images were produced using 'Pymol 2.0'.<br>3. The dimer structure was made using 'Discovery Studios 2.0'<br>4. MALS data were collected and analysed using 'ASTRA 6'<br>5. ITC data were analysed with the MicroCal 'Origin 5.0' software package<br>6. Single-molecule data were first analyzed using custom IDL scripts that allowed automatic extraction of single-molecule time trajectories. Single-molecule trajectories were analyzed using custom MATLAB scripts that allowed manual selection and processing of the trajectories. Statistical analysis was performed using both standard functions in IDL (version 8.2), MATLAB R2017b, OriginPro 2015 (version Sr1 b9.2.257) and Microsoft Excel365. Figure panels for Fig. 1, Fig. 6 and ED Fig. 8 were edited by Adobe Photoshop 2020 and Adobe Illustrator 2020.

For manuscripts utilizing custom algorithms or software that are central to the research but not yet described in published literature, software must be made available to editors/reviewers. We strongly encourage code deposition in a community repository (e.g. GitHub). See the Nature Research guidelines for submitting code & software for further information.

Data

Policy information about availability of data

All manuscripts must include a data availability statement. This statement should provide the following information, where applicable:

- Accession codes, unique identifiers, or web links for publicly available datasets
- A list of figures that have associated raw data
- A description of any restrictions on data availability

The source data underlying Figs 1f-j, 4b–e, 6b, 6d and 6g and Supplementary Figs 1a-f, 2b, 7b-c, 7e and 8c are provided as a Source Data file. Coordinates and structure factors have been deposited in the Protein Data Bank (PDB) with the accession code '6IY8 [https://www.rcsb.org/structure/6IY8]'. Other data are available from the corresponding authors upon reasonable request.

Field-specific reporting

Please select the one below that is the best fit for your research. If you are not sure, read the appropriate sections before making your selection.

- [x] Life sciences
- [ ] Behavioural & social sciences
- [ ] Ecological, evolutionary & environmental sciences

For a reference copy of the document with all sections, see nature.com/documents/nr-reporting-summary-flat.pdf

Life sciences study design

All studies must disclose on these points even when the disclosure is negative.

Sample size	Experiments that yielded a single value per data point were performed as three independent experimental replicates.
Data exclusions	For the single-molecule photobleaching assays, all traces were selected and analyzed that are exhibited by sufficient amount of intensity intially and followed by step-wise decreasing until a basal level intensity. In contrast, traces that displayed compicated (e.g. fluctuation, nothing end at the trajectory) were excluded from the analysis as these molecules may correspond to false positive signals. In some cases attempts to reproduce data on microscope slides of low quality (poor surface-passivation) failed. These datasets were excluded and repeated on high-quality microscope slides.
Replication	To verify the reproducibility, three-five individual experiments were performed and analyzed. We hereby confirm we can reproduce the data presented in this manuscript. All presented data is representative of three-five replicates that yielded similar results.
Randomization	To allow accurate comparison between the data presented in the figures, the substrates that were used for each figure were grouped and the data was obtained at the same day. Randomization is not applicable in this case.
Blinding	Analysis performed in this manuscript were not blinded. However, strict selection criteria were used to ensure reproducibility of the analysis.

Reporting for specific materials, systems and methods

We require information from authors about some types of materials, experimental systems and methods used in many studies. Here, indicate whether each material, system or method listed is relevant to your study. If you are not sure if a list item applies to your research, read the appropriate section before selecting a response.

Materials & experimental systems

n/a	Involved in the study	
[ ]	[x]	Antibodies
[x]	[ ]	Eukaryotic cell lines
[x]	[ ]	Palaeontology
[x]	[ ]	Animals and other organisms
[x]	[ ]	Human research participants
[x]	[ ]	Clinical data

Methods

n/a	Involved in the study	
[x]	[ ]	ChIP-seq
[x]	[ ]	Flow cytometry
[x]	[ ]	MRI-based neuroimaging

Antibodies

Antibodies used	Far-western blot: Anti-His monoclonal antibody (Invitrogen, Catalog #MA1-21315, 3000-fold dilutions) / Anti-mouse lgG (sigma, Catalog #A3562, 30,000-fold dilutions)<br>SMPB: Anti-GFP (biotin) goat polyclonal antibody (Abcam, Catalog #ab6658, 5 pg)
Validation	The web address with validation results is as follows:<br>1.Anti-His monoclonal antibody (Invitrogen, Catalog #MA1-21315)

https://www.thermofisher.com/antibody/product/6x-His-Tag-Antibody-clone-HIS-H8-Monoclonal/MA1-21315
2. Anti-mouse IgG (sigma, Catalog #A3562)
https://www.sigmaaldrich.com/catalog/product/sigma/a3562?lang=ko&region=KR
3. Anti-GFP (biotin) goat polyclonal antibody (Abcam, Catalog #ab6658)
https://www.abcam.com/gfp-antibody-biotin-ab6658.html
